# Wernicke’s encephalopathy in a malnourished surgical patient: a difficult diagnosis

**DOI:** 10.1186/1756-0500-7-718

**Published:** 2014-10-14

**Authors:** Stefano Busani, Cinzia Bonvecchio, Arianna Gaspari, Marcella Malagoli, Alessandra Todeschini, Nicola Cautero, Massimo Girardis

**Affiliations:** Cattedra di Anestesia e Rianimazione, Policlinico di Modena, Università di Modena e Reggio Emilia, Modena, Italy; Servizio di Neuroradiologia, N.O.C.S.A.E., Modena, Italy; Centro Trapianti di Fegato e Multiviscerale, Policlinico di Modena, Modena, Italy

**Keywords:** Wernicke’s encephalopathy, MRI, Septic shock, Cerebral bleeding

## Abstract

**Background:**

Wernicke’s encephalopathy is an acute neurological disorder resulting from thiamine deficiency mainly related to alcohol abuse. Severe thiamine deficiency is an emerging problem in non-alcoholic patients and it may develop in postoperative surgical patients with risk factors.

**Case presentation:**

We reported a case of a 46 years old woman who underwent, one year before, to cephalic duodenopancreatectomy complicated with prolonged recurrent vomiting. She underwent to a second surgical operation for intestinal sub-occlusion and postoperatively she developed septic shock and hemorrhagic Wernicke’s disease. After ICU admission, because of neurological deterioration, she underwent CT scan and MRI that highlighted a strong suspicion for Wernicke’s disease. We treated her with an initially wrong low dose of thiamine, then after MRI we increased the dosage with a neurological status improvement. Despite therapeutic efforts used to control septic shock and thrombocytopenia, she died on the 21^st^ day after surgery because of massive cerebral bleeding and unresponsive cerebral edema.

**Conclusion:**

Early detection of subclinical thiamine deficiency is a difficult task, as symptoms may be nonspecific. Wernicke’s disease remains a clinical diagnosis because there are no specific diagnostic abnormalities revealed in cerebrospinal fluid, electroencephalogram or evoked potentials. About this, the best aid for a correct diagnosis is the clinical suspicion and clinicians should consider the disorder in any patients with unbalanced nutrition, increased metabolism or impaired food absorption. A hallmark of our case was the brain hemorrhage in the typical areas of the Wernicke’s disease, maybe triggered by the thrombocytopenia secondary to sepsis. It might be a good clinical practice administer thiamine to all patients presenting with coma or stupor and risk factors related with thiamine deficiency. Any therapeutic delay may result in permanent neurological damage or death.

## Background

Wernicke’s encephalopathy (WE) is an acute neurological disorder resulting from thiamine deficiency mainly due to alcohol abuse. Other causes reported are: chronic dietetic deficiency, prolonged total parenteral nutrition without thiamine addition, increased nutrients requirements as in trauma or in septic shock [1]. This disease is often under-diagnosed and under-treated being easily confused with other neurological problems especially in critically ill patients. Severe thiamine deficiency is an emerging problem in non-alcoholic patients and it may develop in postoperative surgical patients with risk factors [2].

We reported a case of a malnourished patient who underwent surgery complicated with septic shock and hemorrhagic WE.

## Case presentation

We reported a case of a 46 years old woman admitted to our hospital because of prolonged recurrent vomiting in a severe state of malnutrition. A year before she had been submitted to duodenopancreatectomy because of a stenotic duodenal adenocarcinoma. Immediately, after hospital admission she underwent to a new explorative laparotomy because of worsening symptoms. In this second abdominal surgery she underwent to gastric enteric anastomosis because of sub-occlusion.

Her ability to absorb substances was already compromised by the first operation. During the last year she did not received an adequate nutrition, moreover she had no vitamins supplement in her diet. After the second surgery she was fed by total parenteral nutrition with glucose solutions, amino-acids solutions and vitamins complex (Cernevit – Baxter^@^ spa, Roma, Italy) commonly administered to post-surgical patients containing 3,51 mg of thiamine.

After five days from the gastric enteric anastomosis, she developed a Pseudomonas Aeruginosa’s severe sepsis with acute renal failure; so, she was transferred to our Intensive Care Unit (ICU). At ICU admission, she was agitated but she still had a normal Glasgow Coma Scale (GCS 15/15). Brain Computed Tomography (CT) scan control was negative and altered neurological status of the patient was imputed to the severe sepsis. Her blood tests showed leucopenia (1680/ml) and thrombocytopenia (34000/μl).

We performed an electroencephalogram (EEG) control that evidenced slow diffused anomalies, without paroxysms or focal signs. During the night she was sedated with benzodiazepines and intubated because of respiratory worsening.

**Figure 1:**
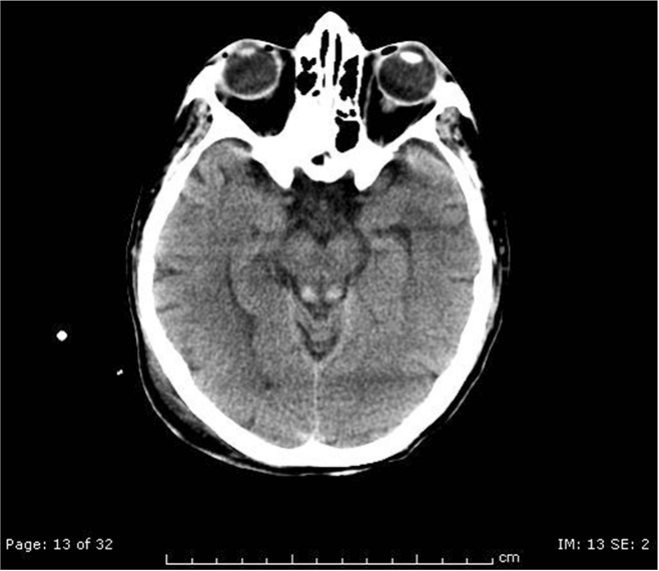
**CT scan shows faded hyperdensity of the mammillary bodies and symmetrical hemorrhagic lesions in the quadrigeminal plate.**

**Figure 2:**
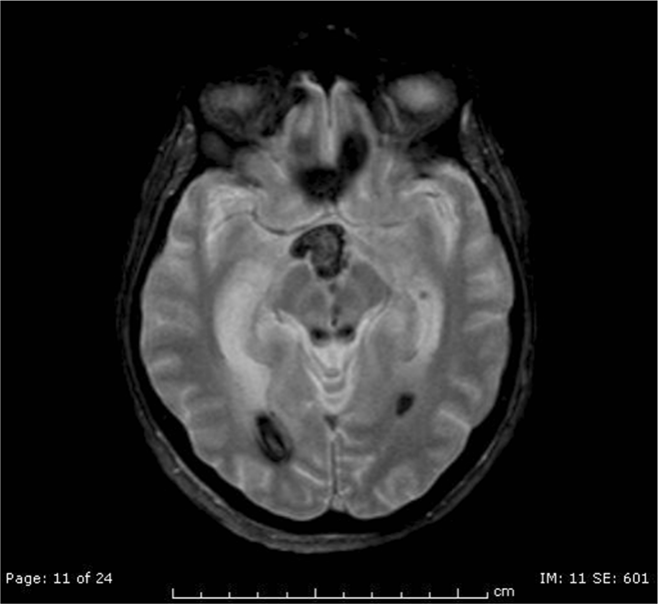
T2* axial shows hemorrhagic necrosis in the quadrigeminal plate and hemorrhagic lesions at the level of mammilllary bodies and optic tracts associated with intraventricular blood clot.

**Figure 3:**
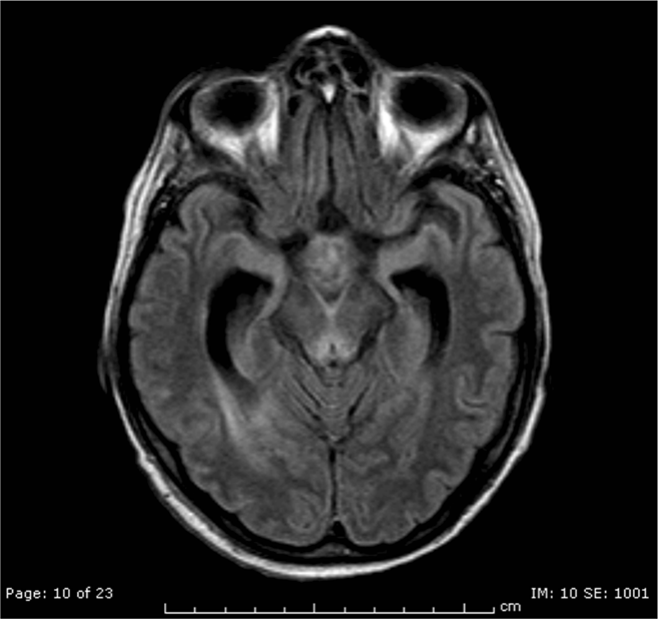
**FLAIR axial shows hyperintensity of the periaqueductal grey.**

**Figure 4:**
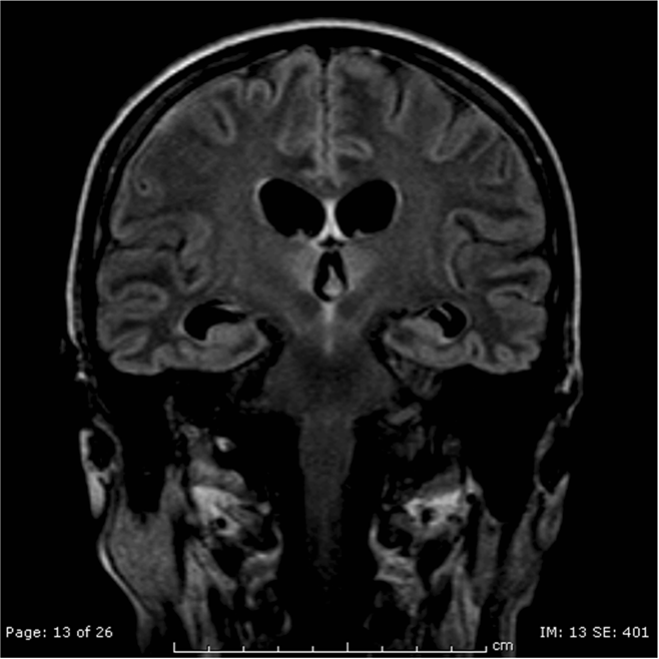
**FLAIR coronal shows hyperintensity of the hypothalamic region and medial thalamus.**

The following day, at the neurological examination she was drowsy, she had no response to pain stimuli, no oculocephalic reflex, limbs areflexia and negative Babinski reflex. A new EEG control pointed out a diffused cerebral suffering state. Simultaneously her renal and liver function got worse due to the evolution of the septic state. Her feeding was still composed by glucose solutions, vitamins (Cernevit – Baxter^@^ spa, Roma, Italy) and amino acid intravenously administered. Then, we decided to perform a new brain CT scan with contrast in order to diagnose the cause of the worsening. This CT scan was performed at day 7 after surgery. The report of the CT scan showed a faded hyperdensity of the mammillary bodies and symmetrical hemorrhagic lesions in the quadrigeminal plate (Figure 1). The report suggested the need of a further diagnostic study with MRI because of the suspicion of a possible metabolic encephalopathy compatible with WE complicated by petechial hemorrhages.After the CT scan, we introduced thiamine 100 mg intravenously once a day, as supplement to the usual feeding. At the following neurological examination her GCS was 7/15, she did not open her eyes, did not emit verbal responses and localized with the right arm to painful stimuli. So 2 days later, we performed a brain magnetic resonance imaging (MRI). The MRI confirmed the lesions described in the CT scan and also added some elements compatible with WE: hyperintensity lesions in T-2 weighted images in the hypothalamus, medial thalamus, periaqueductal grey matter, anatomical regions of the mammillary bodies and superior cerebellar peduncles. (Figures 2, 3 and 4), Mammillary bodies and optic tracts were difficult to assess due to a sub acute blood clot occupying the lower anterior portion of the third ventricle. The study was concluded with the MRI angiography to exclude a malformation of the vessels that had been able to generate the bleeding. Anyway, MRI angiography 3D-TOF was negative for vascular malformations. All these radiological findings were consistent with the diagnosis of WE. In the meantime, patients developed septic shock due to multi-resistant drug Enterococcus Faecium with severe low platelets count needing a mean of 20 Units of platelets transfused per day to maintain a serum level greater than 30000/μl.

No neurosurgical indications were given for the hemorrhagic brain lesions. So, we increased thiamine dosage up to 250 mg intravenously three times per day. This therapy was prolonged for one week, and after 4 days of high-dose thiamine supplementation neurological status of the patient had a significant improvement despite severe septic shock, she was able to open her eyes spontaneously and she performed simple commands.

Nevertheless, septic shock worsened and thrombocytopenia became refractory to platelets transfusions, hemodynamic instability became unresponsive to vasopressors and liver hypoperfusion worsened. Despite severe efforts used to control septic shock and thrombocytopenia, she died on the 21^st^ day after surgery because of massive cerebral bleeding and unresponsive cerebral edema. The macroscopic autopsy of the brain was not nullifying because intracerebral hemorrhage and severe cerebral edema led to colliquation the typical areas of the Wernicke’s disease.

## Discussion

WE is an acute neurologic syndrome resulting from a deficiency in thiamine, which in its biologically active form (thiamine pyrophosphate) is a cofactor in several biochemical pathways in the brain [3].

Early detection of subclinical thiamine deficiency is a difficult task, as symptoms may be nonspecific, such as headaches, fatigue, irritability and abdominal discomfort [4]. Definite thiamine deficiency presents with WE, which has an acute onset and is characterized by mental status changes, ocular abnormalities, and gait ataxia [4]. About 82% of patients have mental status changes according to autopsy-based series [5]. Unfortunately, autoptical brain examination in our case couldn’t conclusively demonstrate the typical histological lesions because of massive hemorrhage in the typical anatomical site and severe swelling.

WE remains a clinical diagnosis because there are no specific abnormalities detectable in cerebrospinal fluid, EEG, or evoked potentials. About this, the best aid for a correct diagnosis is only the clinical suspicion and neurologists or intensivists should consider this disorder in any patients with unbalanced nutrition, increased metabolism or impaired food absorption [6].

We found many risk conditions in our patient: surgical procedures that led to exclusion of portion of the gastrointestinal tract; recurrent vomiting lasting several months associated with intestinal sub-obstruction and malnutrition; systemic disease such as sepsis that affected thiamine intake and metabolism and parenteral nutrition without replacement.

Early diagnosis and treatment of WE is crucial in order to avoid persistent brain damage [6]. The presumptive diagnosis of WE can be confirmed by determining blood thiamine concentrations or by measuring the red blood cell transketolase activity [7]. However, these measurements are limited by a lack of specificity and technical difficulty [8]. Another tool that can help the clinician in WE diagnosis is represented by MRI [9], altered signal in mammillary bodies and periaqueductal grey. A serious complication of WE is secondary hemorrhage, often associated with poor outcome [10]. In our patient, CT scan and MRI have helped the clinician in identifying the Wernicke’s disease, but severe bleeding in the typical sites has altered the anatomy not allowing a definitive diagnosis. Cerebral bleeding was precipitated by the concomitant presence of the severe thrombocytopenia.

Despite the non-diagnostic certainty of neuroradiological images, the sharp improvement in the neurological state of the patient, after the administration of high doses of thiamine, was significant of metabolic disease such as Wernicke.

So, patients who present with symptoms consistent with WE should be empirically treated with a high dose of thiamine hydrochloride three times per day for 2–3 days. In the case there is no response to therapy, supplementation may be discontinued [6]. Doses of thiamine between 100 mg and 250 mg per day apparently may not restore vitamin status, improve clinical signs, or prevent death [6, 11]. Despite these treatments suggestions, there is uncertainty about standard dosage and duration in clinical practice, because they are based only on observational studies and case series [12].

Thiamine treatment in our patient was started later because of severe sepsis that masked early neurological signs. At the beginning, our thiamine treatment was inappropriate because we used a low dosage; only after MRI report we modified the therapy and after few days we observed a strong improvement in GCS. A prompt treatment with thiamine might have saved life to our patient.

Our case report confirms that a WE diagnosis, in non-alcoholic patients, is difficult and early radiological images may not be conclusive if hemorrhage masks the typical lesions.

## Conclusion

What emerges from our experience is that WE has a variable clinical presentation, and it is good practice to treat with thiamine all patients who present in coma or stupor with risk factors associated with thiamine deficiency. The suggestion that we can draw is that such treatment should be undertaken even if neuro-radiological findings are not conclusive. Any therapeutic delay may result in permanent neurological damage or death; conversely, prompt therapy with thiamine may be a life-saving measure.

## Consent section

Written informed consent was obtained from the next of kin of the patient for publication of this case report and any accompanying images. A copy of the written consent is available for review by the Editor of this journal.
